# Supplementary material for: Small-world complex network generation on a digital quantum processor
Source: Nat Commun. 2022 Aug 2;13:4483. doi: 10.1038/s41467-022-32056-y (PMC9345974; doi:10.1038/s41467-022-32056-y)
Supplement: Supplementary file 1 — Supplementary Information [file 41467_2022_32056_MOESM1_ESM.pdf]

Supplementary Information: Small-world complex network generation on a digital quantum processor

Eric B. Jones,[1,2,*] Logan E. Hillberry,[3] Matthew T. Jones,[4,5] Mina Fasihi,[4] Pedram Roushan,[6] Zhang Jiang,[6] Alan Ho,[6] Charles Neill,[6] Eric Ostby,[6] Peter Graf,[1] Eliot Kapit,[4,7,†] and Lincoln D. Carr[4,7,‡]

[1] *National Renewable Energy Laboratory, Golden, CO 80401, USA*
[2] *ColdQuanta Inc., Boulder, CO 80301, USA*
[3] *Department of Physics, University of Texas, Austin, TX 78712, USA*
[4] *Department of Physics, Colorado School of Mines, Golden, CO 80401, USA*
[5] *NVIDIA Corporation, Boulder, CO 80302, USA*
[6] *Google Quantum AI, Santa Barbara, CA 93117, USA*
[7] *Quantum Engineering Program, Colorado School of Mines, Golden, CO 80401, USA*

(Dated: June 2, 2022)

CONTENTS

Supplementary Note 1 – Quantum Cellular Automata in 1D 1

Supplementary Note 2 – Quantum Hardware Specifications and Qubit Picking 2
  Weber Processor 2
  Rainbow Processor 3

Supplementary Note 3 – Circuit Compilation, Calibration, and Optimization 4

Supplementary Note 4 – Post-Selection 6

Supplementary Note 5 – Mutual Information Measures 8

Supplementary Note 6 – Complex Network Measures 11
  Clustering Coefficient 11
  Average Shortest Path Length 12
  Node-Strength Distribution 12
  Relationship to Prior Complex Mutual Information Network Analyses 13

Supplementary Note 7 – Establishing the Coherence Window 13

Supplementary Note 8 – Effect of Higher Product State Filling 14

Supplementary Note 9 – Comment on Simulation Platform Choice 14

References 15

SUPPLEMENTARY NOTE 1 – QUANTUM CELLULAR AUTOMATA IN 1D

A one-dimensional quantum cellular automaton is defined as a chain of L identical qubits whose states are updated according to homogeneous blocks of neighborhood-local unitary operators [1, 2]. Each local unitary takes as an input the state of the target qubit's neighbors and outputs a particular operator, either the identity or the activation operator, to be applied to the target qubit, conditioned on the neighborhood's state. When such an update unitary has been applied to all L qubits, a QCA *cycle* is complete. In the specific construction with nearest-neighbor connectivity, corresponding to three-site (closed) neighborhoods (denoted T_R), a specific qubit's open neighborhood (its two neighbors) can be in a superposition of any 2^2 states. The target qubit's state can either be activated or not depending upon these 4 configurations. As such, there are $2^{2^2} = 16$ possible transition functions on a three-site closed neighborhood. We label each of these transition functions, or *rules*, as $R \in \{0, 1, \ldots, 15\}$. To specify a three-site rule, we write T_R. The rule which activates for only a balanced neighborhood of 0-1 or 1-0, T_6, is called the *Goldilocks rule* and is the main focus of our study on the quantum processor.

For efficient circuit parallelization purposes, we update all even qubits in parallel followed by all odd qubits. In this ordering, the general form for the L-qubit unitary operator that evolves the system from cycle t to cycle $t+1$ is [3]

$$\begin{aligned} U(T_R; t, t{+}1) &= \prod_{o=1,3,\ldots}^{L} U_o(T_R) \prod_{e=2,4,\ldots}^{L-1} U_e(T_R), \\ U_{e(o)}(T_R) &= \sum_{m,n=0}^{1} P_{e(o)-1}^{(m)} \otimes V_{e(o)}^{c_{mn}} \otimes P_{e(o)+1}^{(n)} \end{aligned} \tag{1}$$

where $i = e(o)$ indexes each even (odd) qubit in the 1D chain, $P_j^{(m)} = |m\rangle\langle m|$ is a single-qubit projection operator, and V_i is the chosen activation unitary applied to qubit i (which is typically taken to be the same operator for all i). In order to convert from a rule number to a unitary operator, one first performs a binary expansion of the rule number where the expansion coefficients are themselves labelled by 2-bit strings, $R = \sum_{m,n=0}^{1} c_{mn} 2^{m+n}$. The coefficients c_{mn} describe

which neighborhood configurations activation occurs on. For Goldilocks rule T_6 considered in the main text, we have that $6 = 1\times 2^1 + 1\times 2^2$ so that $c_{00} = c_{11} = 0$ while $c_{01} = c_{10} = 1$. Therefore, the local T_6 cycle unitary reads

$$U_i(T_6) = |00\rangle 1\langle 00| + |01\rangle V\langle 01| + |10\rangle V\langle 10| + |11\rangle 1\langle 11|, \quad (2)$$

which applies the activation unitary V to qubit i if its surrounding qubits are in the configurations $|01\rangle$ or $|10\rangle$ and does nothing otherwise. We refer to T_6 as a *totalistic* rule because activation obeys a left-right symmetry and only depends on the total number of adjacent $|1\rangle$s. Another important totalistic rule is T_1, also called the PXP model in many-body quantum literature when run in continuous time [3]; here we consider discrete time, i.e., a quantum circuit, in line with the main concept of cellular automata. Note that that for 1D, three-site neighborhoods there are only $2^3 = 8$ totalistic rules. Restricting a QCA search space to require totalistic updates is a useful tool for discovering emergent complexity. Finally, we note that $V = X$, the bit-flip operator, corresponds to a reversible classical cellular automaton. In the main text we choose $V = H$, the Hadamard operator. It has been shown that the particular form of the activation unitary affects the complexity outcomes of T_6 relatively little so long as V is able to move classical states off the poles of the Bloch sphere, which H does [3].

The continuous time versions of the QCA considered herein, of which the T_6 rule is a specific instance, evolve according to *kinetically-constrained* Hamiltonians. Models of this type were originally introduced to study the dynamics of glassy systems [4]. Their characteristic attribute is that degrees of freedom evolve explicitly as a function of the state of their neighborhood– this can be seen as embodied via the projection operators in Eq. 1. Given that kinetically-constrained models were originally introduced to study glassy dynamics, it is a reasonable question as to whether the T_6 QCA that we simulate corresponds to some spin glass-related model that has already been simulated on a gate-model quantum processor. As demonstrated in Section V.A. of [3], the continuous time, *analog* T_6 QCA can be *approximated* by a transverse-field Ising model (TFIM) with a very particular structure. However, there are a few important ways in which the discrete time simulation of rule T_6 differs from (and therefore is not simply equivalent to) Trotterized evolution of the TFIM. First, while the discrete time QCA cycle unitary $U(T_6; t, t+1)$ commutes with the dynamical invariant operator used for post-selection, $\mathcal{O}$, the TFIM Hamiltonian does not. Hence, the two systems have different conservation laws, although for certain discrete time steps it is the case that the TFIM evolution unitary does commute with $\mathcal{O}$ [5]. Second, using the TFIM to model the dynamics of the continuous time T_6 rule only works when the Hermitian activation generator is the Pauli X operator. However, our activation unitary is the Hadamard operator, $V_j = H$, which cannot be generated by X alone. Finally, even if some slight alteration of the TFIM could generate the local Hadamard activation unitary, say via $V_j = \exp(-i\alpha H)$, this would correspond to a Trotter step of $\alpha = \pi/2$, which cannot be regarded as an accurate Trotterization of the continuous limit. Therefore, the discrete time T_6 QCA considered in this work cannot simply be mapped onto the TFIM. Since we have ruled out the TFIM as the appropriate Trotterized model Hamiltonian corresponding to the discrete time T_6 QCA, and since to our knowledge no other kintetically-constrained Hamiltonian has been simulated on a digital quantum processor, we believe the present work to constitute the first quantum simulation of a QCA on a gate-model quantum computer.

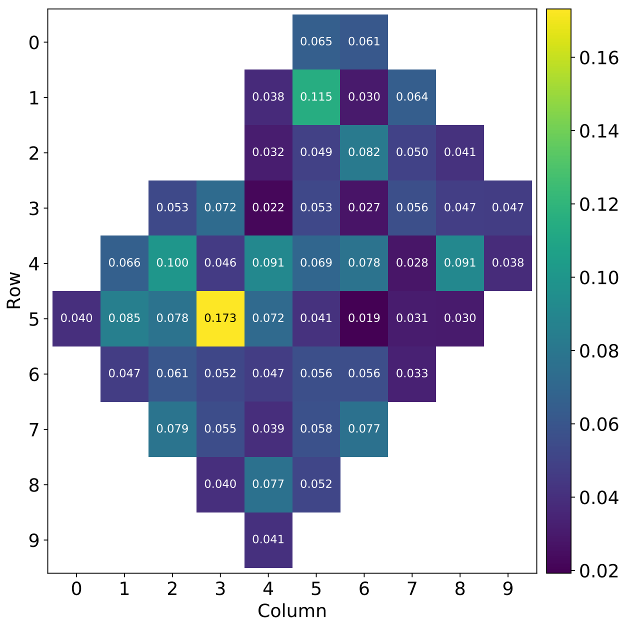

Supplementary Figure 1. **Weber parallel $|1\rangle$-state readout error rates.** Each qubit is represented by a square and is indexed by a row and column number. Heatmap values represent the probability of finding a qubit in the $|0\rangle$ state after it was prepared in the $|1\rangle$ state when all qubits are read out in parallel.

SUPPLEMENTARY NOTE 2 – QUANTUM HARDWARE SPECIFICATIONS AND QUBIT PICKING

Weber Processor

The Weber quantum processing unit is a 53-qubit superconducting processor that follows the design of the Sycamore-class architecture in Ref. [6]. As discussed in the main text, typical performance characteristics for

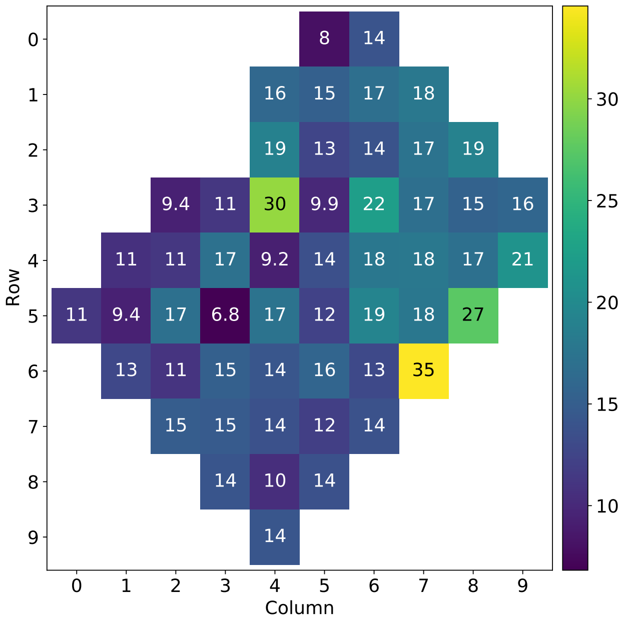

Supplementary Figure 2. **Weber idle $\tilde{T}_1$ decoherence times (μs).** Each qubit is represented by a square and is indexed by a row and column number. Heatmap values represent the idle $\tilde{T}_1$ decoherence time in μs.

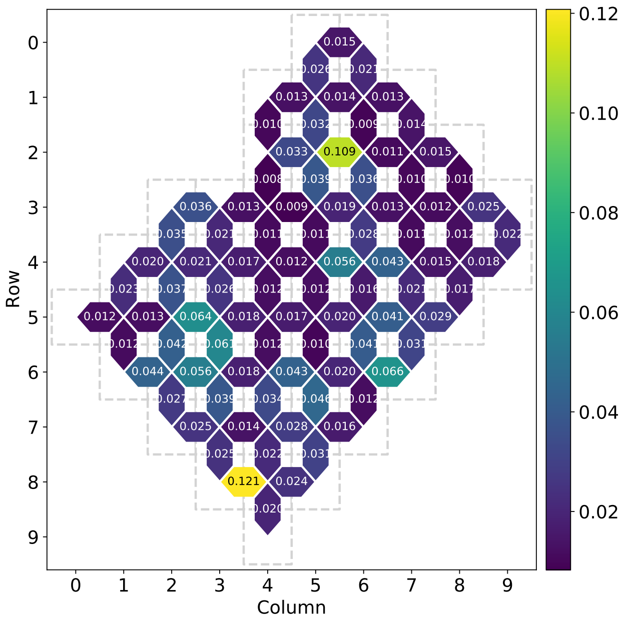

Supplementary Figure 3. **Weber parallel $\sqrt{\text{iSWAP}}^\dagger$ Pauli error rates.** Each qubit is indexed by a row and column number, and hexagons represent couplers. Heatmap values represent the cross-entropy benchmarking Pauli error per cycle when executed in parallel (see [6]).

Weber are: single-qubit gate error $e_1 \approx 0.1\%$, two-qubit gate error $e_2 \approx 1.4\%$, $|0\rangle$-state readout error $e_{r0} \approx 2\%$, $|1\rangle$-state readout error $e_{r1} \approx 7\%$, and population relaxation time $\tilde{T}_1 \approx 15\mu s$ [7]. Of these, the three performance characteristics that are potentially the most deleterious to calculating accurate observables are e_{r1}, e_2, and $\tilde{T}_1$ (relative to total circuit execution time). Selecting high-quality chains of qubits therefore involves simultaneously optimizing for these three characteristics, which change slightly between processor calibrations.

For the particular calibration displayed, Supplementary Figure 1 shows that while the parallel readout e_{r1} on Weber is relatively homogeneous across the chip, it is slightly lower on the right-hand side. Meanwhile, the idle $\tilde{T}_1$ decoherence times shown in Supplementary Figure 2 are somewhat longer on the right-hand side of the processor as well and relatively uniform within its top right quadrant. Finally, e_2 rates for the $\sqrt{\text{iSWAP}}^\dagger$ gate when executed in parallel on Weber and characterized by cross-entropy benchmarking (XEB) are lower both in the processor's top-right quadrant and in a small region left of center. Taken together, these observations indicate that the top-right quadrant of Weber is an ideal region within which to choose 1D qubit embeddings. For instance, a particular five qubit embedding that exploits this quadrant has qubit indices (1, 6) $\rightarrow$ (2, 6) $\rightarrow$ (2, 7) $\rightarrow$ (3, 7) $\rightarrow$ (4, 7). Of course, as chains become longer, that is, as L becomes larger, the available real-estate for picking the highest-quality qubits becomes constrained.

Rainbow Processor

The Rainbow quantum processing unit is a 23-qubit superconducting processor with typical performance characteristics similar to Weber. While the results in the main text were generated on the Weber processor, we used Rainbow in order to assess the effect of varying initial conditions in the product state. These varying initial conditions were assessed in a 17-qubit chain, the largest 1D chain embeddable on Rainbow. This size of the chain is large enough to not be susceptible to the finite-size effects encountered in smaller chains. At the same time it is not so large that low retained count fractions cause problems with measurement statistics and thus create large error bars. Figs. 4, 5, and 6 show the three most relevant performance characteristics for choosing high-quality chains, parallel e_{r1}, $\tilde{T}_1$, and parallel e_2, respectively. However, the ability to avoid noisy qubits on Rainbow was severely constrained by the large size of the chain relative to the size of the processor. For reference, we used the embedding (5, 0) $\rightarrow$ (5, 1) $\rightarrow$ (4, 1) $\rightarrow$ (4, 2) $\rightarrow$ (4, 3) $\rightarrow$ (5, 3) $\rightarrow$ (5, 2) $\rightarrow$ (6, 2) $\rightarrow$ (7, 2) $\rightarrow$ (7, 3) $\rightarrow$ (6, 3) $\rightarrow$ (6, 4) $\rightarrow$ (6, 5) $\rightarrow$ (7, 5) $\rightarrow$ (7, 4) $\rightarrow$ (8, 4) $\rightarrow$ (8, 5).

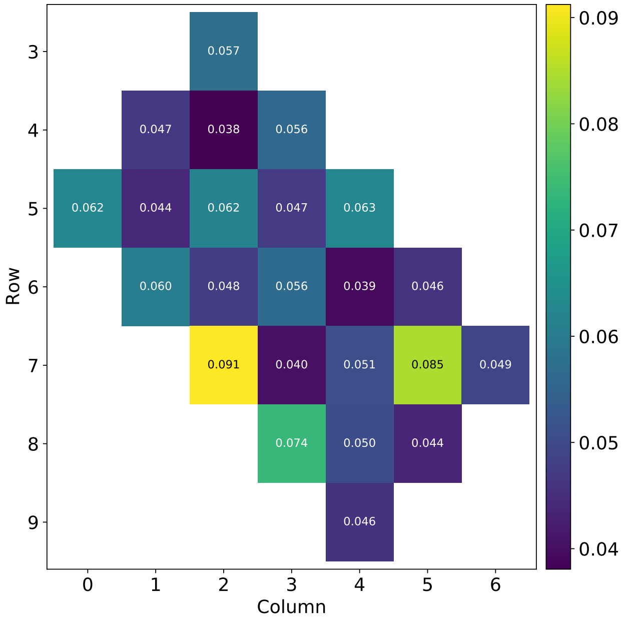

Supplementary Figure 4. **Rainbow parallel $|1\rangle$-state readout error rates.** Same data as Supplementary Figure 1, but for the 23-qubit Rainbow quantum processor.

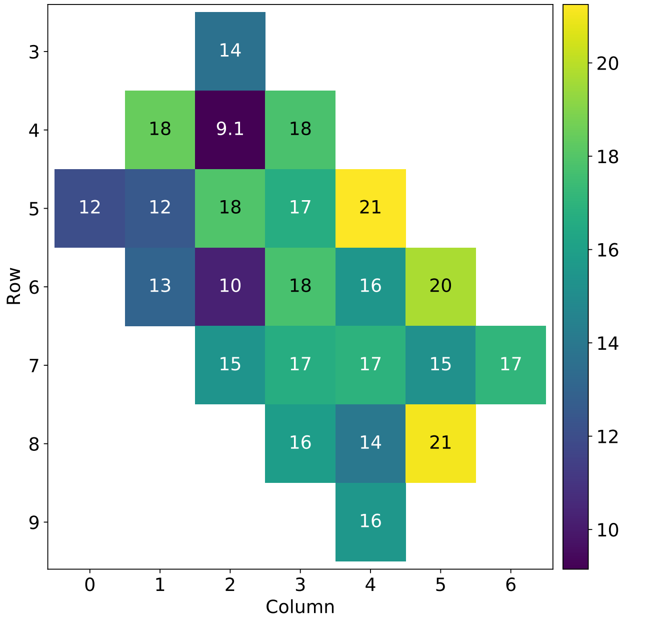

Supplementary Figure 5. **Rainbow idle $\tilde{T}_1$ decoherence times.** Same data as Supplementary Figure 2, but for the 23-qubit Rainbow quantum processor.

SUPPLEMENTARY NOTE 3 – CIRCUIT COMPILATION, CALIBRATION, AND OPTIMIZATION

In order to simulate the T_6 QCA with high-fidelity, we compile, calibrate, and optimize circuits in the following manner. The Cirq open source framework was used for the workflow [8].

1. Each $CH(q_i, q_j)$ gate between control qubit q_i and target qubit q_j in the local T_6 update unitary is compiled into

$$CH(q_i, q_j) = Y^{1/4}(q_j) CZ(q_i, q_j) Y^{-1/4}(q_j), \qquad (3)$$

where up to a global phase $Y^t = R_Y(\pi t)$.

2. Each CZ gate is further decomposed into two bare $\sqrt{\text{iSWAP}}^\dagger$ gates and single-qubit rotations. A controlled-phase gate between control qubit q_i and target qubit q_j can be decomposed as

$$\begin{aligned}\text{CPHASE}(\phi)_{ij} = e^{i(2\varphi-\phi)/4} & \\ \times R_{Z_i}(\pi - \phi/2) \otimes R_{Z_j}(-\phi/2) & \\ \times R_{X_i}(-\xi_i) \otimes R_{X_j}(-\xi_j) & \\ \times K(\theta)_{ij}\text{CPHASE}(\varphi)_{ij} & \\ \times R_{Z_i}(\pi + \varphi/2) \otimes R_{Z_j}(\varphi/2) & \qquad (4) \\ \times R_{X_i}(-2\alpha) \otimes 1_j & \\ \times K(\theta)_{ij}\text{CPHASE}(\varphi)_{ij} & \\ \times R_{Z_i}(\phi/2) \otimes R_{Z_j}(\phi/2) & \\ \times R_{X_i}(\xi_i) \otimes R_{X_j}(\xi_j), &\end{aligned}$$

which is equivalent to the decomposition presented in Ref. [9]. Here, $K(\theta)$ is a continuously-parameterized fractional-iSWAP gate such that $\sqrt{\text{iSWAP}}^\dagger = K(\pi/4)$. In this step, we take $\varphi = 0$ and account for the parasitic $\varphi \approx \pi/23$ using Floquet calibration in a later step. The other decomposition parameters are given by

$$\sin(\alpha) = \sqrt{\frac{\sin^2(\phi/4) - \sin^2(\varphi/2)}{\sin^2(\theta) - \sin^2(\varphi/2)}}, \qquad (5)$$

$$\begin{aligned}\xi_i = \tan^{-1}\left(\frac{\tan(\alpha)\cos(\theta)}{\cos(\varphi/2)}\right) & \\ + \frac{\pi}{2}\left(1 - \text{sgn}\Big(\cos(\varphi/2)\Big)\right), & \qquad (6)\end{aligned}$$

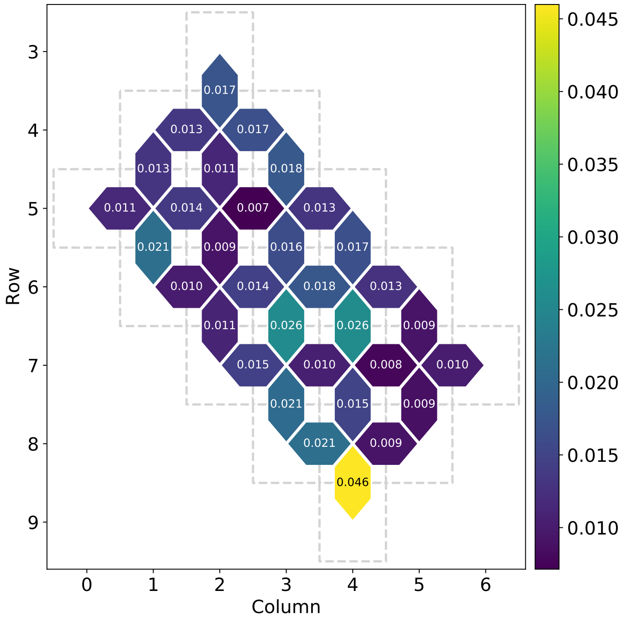

Supplementary Figure 6. **Rainbow parallel $\sqrt{\mathbf{iSWAP}}^{\dagger}$ Pauli error rates.** Same data as Supplementary Figure 3, but for the 23-qubit Rainbow quantum processor.

and

$$\xi_j = \tan^{-1}\left(\frac{\tan(\alpha)\sin(\theta)}{\sin(\varphi/2)}\right) + \frac{\pi}{2}\Big(1 - \mathrm{sgn}\Big(\sin(\varphi/2)\Big)\Big). \tag{7}$$

To decompose the CZ gate, we simply set $\phi = \pi$.

3. Strings of consecutive single-qubit gates are merged together into $\mathrm{PhXZ}(a, x, z) \equiv Z^z Z^a X^x Z^{-a}$ gates using the cirq.google *optimized_for_sycamore* utility. This compresses into alternating moments of parallel single-qubit gates followed by parallel two-qubit gates.

4. Spin echo insertion: Wherever a qubit is idle for more than one single-qubit gate layer, pairs of Pauli operators are inserted to decrease idle qubit crosstalk. For instance, $XX = 1$.

5. Floquet characterization: Our targeted native two-qubit gate on Weber is the $\sqrt{\mathrm{iSWAP}}^{\dagger} = K(\pi/4)$ gate. However, due to calibration drift and cross-talk errors, the effective gate implemented has the form (see Supplementary Information of Refs. [9, 10])

$$K(\theta, \zeta, \chi, \gamma, \varphi) = \begin{bmatrix} 1 & 0 & 0 & 0 \\ 0 & e^{-i\gamma - i\zeta}\cos(\theta) & -ie^{-i\gamma + i\chi}\sin(\theta) & 0 \\ 0 & -ie^{-i\chi - i\gamma}\sin(\theta) & e^{-i\gamma + i\zeta}\cos(\theta) & 0 \\ 0 & 0 & 0 & e^{-2i\gamma - i\varphi} \end{bmatrix}. \tag{8}$$

In the absence of calibration drift and cross-talk errors, $\theta = \pi/4$ and $\zeta = \chi = \gamma = \varphi = 0$, and one recovers the target gate as $\sqrt{\mathrm{iSWAP}}^{\dagger} = K(\pi/4, 0, 0, 0, 0)$. When such drift and errors are present, the goal of Floquet characterization is to determine the values of $\theta, \zeta, \chi, \gamma$, and φ with a view towards subsequently correcting for some or all of them. This is done by repeating circuit moments with two-qubit gates many times with interleaved, parameterized z-rotations as probes, in order to amplify the calibration drift and control errors embodied in $\theta, \zeta, \chi, \gamma$, and φ. All five angles except for χ are able to be characterized, since χ corresponds to a complex hopping phase and requires closed loops in order to characterize. For more details, see Refs. [9, 10].

6. After Floquet characterization, the first angle we correct for is the parasitic cphase angle, φ. We do this by reconstructing our circuits and re-decomposing our CZ gates using Eq. 4 where now instead of $\varphi = 0$ we use the value determined by Floquet characterization on each moment, which is typically in the vicinity of $\varphi \approx \pi/23$.

7. Next, we re-merge single-qubit gates according to the procedure in Step 3.

8. Re-insert spin echoes according to the logic of Step 4.

9. Floquet calibration: In addition to φ, the angles γ and ζ can be corrected for by inserting z-rotations on either side of an imperfect two-qubit gate. The relationship between the general excitation-number-conserving gate and our target gate (with parasitic cphase) is

$$K(\theta, 0, 0, 0, \varphi) = R_Z(-\beta, \beta) R_Z(\gamma, \gamma) K(\theta, \zeta, \chi, \gamma, \varphi) R_Z(-\alpha, \alpha), \tag{9}$$

where $\alpha = (\zeta + \chi)/2$, $\beta = (\zeta - \chi)/2$, and we use the following shorthand for pairs of two single-qubit z-rotations, $R_Z(z_i, z_j) = e^{i(z_i + z_j)/2} R_{Z_i}(z_i) \otimes R_{Z_j}(z_j)$. Since we cannot characterize χ, we set $\chi = 0$. The resulting simplification in Eq. 9 will correct for γ and ζ. As such, after the insertion of the z-rotations in Eq. 9, each CZ gate in the original QCA circuit

is compiled correctly as in Eq. 4 with the effective two-qubit native gate $\sim K(\pi/4, 0, 0, 0, \pi/23) = K(\pi/4)\mathrm{CPHASE}(\pi/23)$. The angles $\theta \sim \pi/4$ and $\chi \sim 0$ remain uncorrected. The inserted, corrective z-rotations are merged with the execution of two-qubit gates at the hardware level and do not need to be re-merged into PhXZ gates.

10. Send circuits to the quantum processor, Weber or Rainbow, in temporal batches, averaging over qubit arrangements.

SUPPLEMENTARY NOTE 4 – POST-SELECTION

Once measurement statistics are gathered by measuring N_c times in the z-basis, post-selection is used to discard measurements, i.e. bit strings, that result from error and thus reside outside the invariant-protected sector of Hilbert space. We emphasize that with a higher fidelity, post-selection would be unnecessary for the establishment of coherence windows; generally however, in the NISQ era post-selection is an important and useful tool for quantum computing. The dynamical invariant that defines the protected sector of Hilbert space is the operator

$$\begin{aligned} \mathcal{O} &\equiv \sum_{i=0}^{L} \mathcal{O}_i, \\ \mathcal{O}_i &\equiv Z_i Z_{i+1} \end{aligned} \tag{10}$$

where the sites $i = 0$ and $i = L + 1$ refer to padding boundary qubits fixed to the $|0\rangle$ state. We first give a proof that $\mathcal{O}$ is a dynamical invariant of the T_6 rule. Consider the commutator between Eq. 1 and Eq. 10

$$\begin{aligned} \Big[\mathcal{O}, U(T_R; t, t+1)\Big] &= \sum_{i=0}^{L} \Big[\mathcal{O}_i, \prod_o U_o \prod_e U_e\Big] \\ = \sum_{i=0}^{L} \Big(\Big[\mathcal{O}_i, \prod_o U_o\Big] \prod_e U_e &+ \prod_o U_o \Big[\mathcal{O}_i, \prod_e U_e\Big]\Big). \end{aligned} \tag{11}$$

Using standard commutator identities, the two commutators in the second line of Eq. 11 can be expressed as

$$\begin{aligned} \Big[\mathcal{O}_i, \prod_j U_j\Big] &= \sum_j \prod_{l'>j} U_{l'} \Big[\mathcal{O}_i, U_j\Big] \prod_{l<j} U_l \\ = \sum_j \prod_{l'>j} U_{l'} \Big(Z_i \Big[Z_{i+1}, U_j\Big] &+ \Big[Z_i, U_j\Big] Z_{i+1}\Big) \prod_{l<j} U_l, \end{aligned} \tag{12}$$

where j can run over either the even or odd index set. Next, note that since any projection operator commutes with the Pauli Z operator,

$$\Big[Z_k, U_j\Big] = P_{j-1}^{(m)} \Big[Z_j, V_j^{c_{mn}}\Big] \delta_{k,j} P_{j+1}^{(n)}. \tag{13}$$

Substituting Eqs. 13 and 12 into Eq. 11 and noting that $Z_k P_k^{(m)} = P_k^{(m)} Z_k = (-1)^m P_k^{(m)}$ we find

$$\begin{aligned} \Big[\mathcal{O}, U(T_R; t, t+1)\Big] = \\ \sum_o \prod_{l'>o} U_{l'} \sum_{m,n} P_{o-1}^{(m)} \Big[Z_o, V_o^{c_{mn}}\Big] P_{o+1}^{(n)} \Big((-1)^m \\ +(-1)^n\Big) \prod_{l<o} U_l \prod_e U_e \\ + \prod_o U_o \sum_e \prod_{l'>e} U_{l'} \sum_{m,n} P_{e-1}^{(m)} \Big[Z_e, V_e^{c_{mn}}\Big] P_{e+1}^{(n)} \Big((-1)^m \\ +(-1)^n\Big) \prod_{l<e} U_l \end{aligned} \tag{14}$$

We now specify to the rule T_6. There are four combinations of m and n to consider. In the instance where $m = n = 0$ or $m = n = 1$, the commutator $[Z_j, V_j^{c_{m=n=0}}] = 0$. Meanwhile, when $m \neq n$, $(-1)^m + (-1)^n = 0$. Therefore, each term indexed by (m, n) vanishes and

$$\Big[\mathcal{O}, U(T_6; t, t+1)\Big] = 0, \tag{15}$$

proving that $\mathcal{O}$ is a dynamical invariant of rule T_6 for *any* unitary V_j.

In addition, $\mathcal{O}$ has eigenstates diagonal in the z-basis. Hence, any z-basis measurement whose eigenvalue under $\mathcal{O}$ is different than the $\mathcal{O}$-eigenvalue of the QCA's initialized state must have resulted from an error in the computation. In order to see this, and that Eq. 10 is related to domain wall conservation, consider the action of $\mathcal{O}$ on a five-qubit chain, initialized with a single, central bit flip, and padded by fixed boundary $|0\rangle$s

$$\mathcal{O}\,|0\rangle \otimes |00100\rangle \otimes |0\rangle = 2\,|0\rangle \otimes |00100\rangle \otimes |0\rangle. \tag{16}$$

Now consider the action of $\mathcal{O}$ on another state with the same number of domain walls

$$\mathcal{O}\,|0\rangle \otimes |01110\rangle \otimes |0\rangle = 2\,|0\rangle \otimes |01110\rangle \otimes |0\rangle. \tag{17}$$

If however a bit flip were to occur on the central qubit in the chain, the action of $\mathcal{O}$ would be

$$\mathcal{O}\,|0\rangle \otimes |01010\rangle \otimes |0\rangle = -2\,|0\rangle \otimes |01010\rangle \otimes |0\rangle. \tag{18}$$

Hence, the calculated eigenvalue of -2, rather than $+2$, indicates that we should discard this measurement from our statistics when calculating observables.

Note that $\mathcal{O}$ does not protect against all longitudinal relaxation errors. For example, if the state in Eq. 17 relaxes on the qubit just left or right of center, $\mathcal{O}$ still has eigenvalue $+2$. In addition, if two photons are lost, say in both the central qubit and in the one just to the right or left, $\mathcal{O}$ will also have eigenvalue $+2$. In instances such as these, superposition amplitudes will renormalize in such

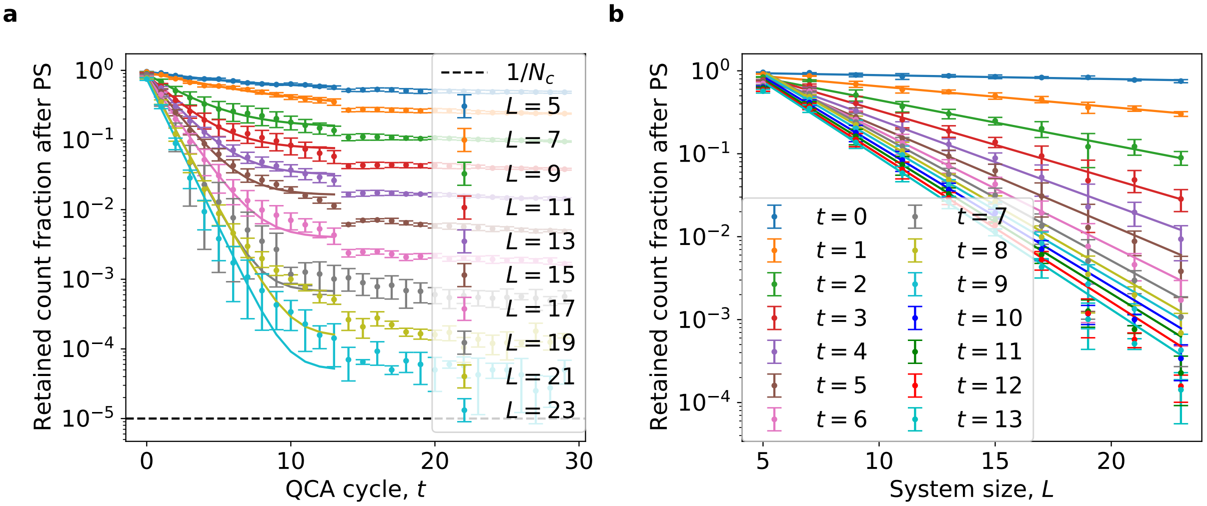

Supplementary Figure 7. **Fraction of z-basis measurements retained after post-selection.** **a** Retained count fraction as a function of QCA cycle for the different system sizes, $L = 5, 7, \dots, 23$. Points represent experimental values, averaged over four qubit chains (embeddings) while the associated error bars represent one standard deviation from the mean. Solid lines represent the function $a_t(L)\exp(-b_t(L)t) + c_t(L)$, fit to data from the first 13 QCA cycles, i.e., before the observed discontinuity in the retained count fractions. The black dashed line signifies $1/N_c$, the minimum, non-zero number of retained counts possible. **b** Retained count fraction as a function of system size for the first 13 QCA cycles. Points represent experimental values, averaged over four qubit chains (embeddings) while the associated error bars represent one standard deviation from the mean. Solid lines represent the function $a_L(t)\exp(-b_L(t)L)$, fit to data from $L = 9$ to $L = 23$, so as to exclude some finite-size effects observed at $L = 5$ and $L = 7$.

a way as to degrade the fidelity of the computation while still keeping the processor's state superposition in the protected subspace.

Figs. 7a-b show the fraction of initial processor counts, N_c, that are retained after applying post-selection to our experimental measurements from Weber as a function of QCA cycle depth for all system sizes considered and as a function of system size for all cycle depths $t \in \{0, 1, \dots, 13\}$. Unsurprisingly, from $t = 0$ to $t = 13$, each set of data points with constant L decays exponentially as a function of QCA cycle as the post-selection procedure has to discard exponentially many measurements in order to compensate for $\tilde{T}_1$ decoherence. At $t = 14$, there is an evident step change in the decay rate of the retained count fractions. The nominal gate execution times for PhXZ and $\sqrt{\text{iSWAP}}^\dagger$ on Weber are 25 ns and 32 ns, respectively. Since each QCA cycle has eight alternating layers of each, at $t = 14$, the nominal circuit execution time (excluding readout) is about 6.384μs. While median $\tilde{T}_1$ times are about 15 μs, low end (10th percentile) $\tilde{T}_1$ times are about 11 μs on Weber. Hence, at 6.384 μs, the probability of a 10th percentile qubit erroneously relaxing from the $|1\rangle$ state is about 44 %, making it difficult for post-selection to compensate for multi-photon loss processes or delocalizing errors. QCA cycle $t = 14$ is also near the point referenced in the main text (e.g., main text Fig. 2d) where the error bars on population dynamics begin to grow significantly.

In addition, the retained count fraction also decays roughly exponentially at fixed t as a function of L. The number of gate layers, and thus the circuit execution time, is fixed as a function of system size for fixed QCA cycle depth with respect to $\tilde{T}_1$. Since gate *volume* however grows linearly in L, it is likely that this decay in the retained count fraction as a function of system size is a result of accumulating gate error, as is well-established within the digital error model [6].

Because the combined effects of noise and post-selection require that exponentially-many measurements are discarded as both a function of system size and cycle depth, we consider the effect this has on the computational complexity of simulating the T_6 QCA on the Sycamore-class processors relative to the computational complexity of emulating the T_6 rule using standard classical algorithms. For reference, the main text already provides a qualitative discussion regarding the use of tensor network approaches for emulating QCA. For a general quantum circuit of L qubits and M gates, the Schrödinger emulation algorithm requires $\sim 2^L$ spatial resources and $\sim M2^L$ temporal resources. The Feynman algorithm requires $\sim M + L$ space and $\sim 4^M$ time [11]. However, the observable $\mathcal{O}$ is also conserved for classical emulation. Therefore, in classical emulations one can use a "domain conserving" basis (see below and Supplementary Figure 8) that reduces the Schrödinger requirements to $\sim L^{1.91}$ (space) and $\sim ML^{1.91}$ (time). Although hybrid Schrödinger-Feynman algorithms also exist for quantum emulation, we focus on comparison

with the Schrödinger algorithm for simplicity and because the large two-qubit gate count of our largest circuits, $M_{2Q} > 1,000$, makes effective utilization of the Feyman algorithm difficult.

As shown in Supplementary Figure 7b, the number of retained counts after post-selection at fixed cycle depth, t, scales as $N_r(t) \sim e^{-b_L(t)L}$ as a function of system size. Since the error, due to shot noise, in calculating a local observable, A, using quantum computer measurements scales as $\langle A\rangle \sim 1/\sqrt{N_r}$, one would need to scale the initial number of counts as $N_c \sim e^{b_L(t)L}$ in order to offset the fraction discarded by post-selection and thus achieve constant precision (We note briefly that we did not follow this methodology in our experimental setup in favor of constant run-time). Therefore, in order to simulate the T_6 QCA on a quantum processor at fixed precision, one would require L spatial resources (qubits) and $\sim d(M(t))e^{b_L(t)L}$ temporal resources. $d(M(t))$ accounts for the fact that gates are often layered in parallel on the processor and that the total gate volume depends upon the desired QCA cycle depth. But the most important element for comparing to the performance of the Schrödinger emulator is the factor $e^{b_L(t)L}$. For the fits shown in Supplementary Figure 7b, $b_L(t) \lesssim 0.422$ over the set $t \in \{0, 1, \ldots, 13\}$. Hence, $e^{b_L(t)L} \lesssim 1.525^L$. The fit parameters $b_L(t)$ depend upon the error rates in the processor, which will generally improve over time, requiring fewer counts to be discarded at each cycle depth. However, in the absence of fault-tolerant, code-based error correction, the time complexity due to post-selection will remain exponential. In summary, quantum simulation of the T_6 rule obtains a nearly quadratic reduction in spatial computational resources as compared to Schrödinger emulation. In exchange, quantum simulation requires exponential time due to post-selection, while classical time complexity scales polynomially using the Schrödinger algorithm with a domain-conserving basis set.

Since T_6 conserves $\mathcal{O}$, the system evolves in a Hilbert space of reduced dimension $\mathcal{H}_{\mathcal{O}}$. In Supplementary Figure 8 we plot this dimension $\dim(\mathcal{H}_{\mathcal{O}})$ as a function of system size for initial conditions consisting of a few $|1\rangle$'s separated by $|0\rangle$'s. For the case studied in the main text, a single $|1\rangle$ initialized in the center of each chain, the protected Hilbert space dimension grows as $\mathcal{H}_{L-3} \sim 0.63\, L^{1.91}$. Stricly-speaking, this scaling in the protected Hilbert space dimension is a bound on the system dynamics. In order to assess the extent to which the T_6 QCA's dynamics actually saturate this bound, Supplementary Figure 9a shows the number of computational basis states occupied during the dynamics as a function of QCA cycle for the different system sizes (solid lines). QCA data is here generated by classical emulation. Dashed lines demarcate the $\dim(\mathcal{H}_{\mathcal{O}})$ scaling bounds. As can be seen over the first 30 QCA cycles, there always appears to be at least one point where

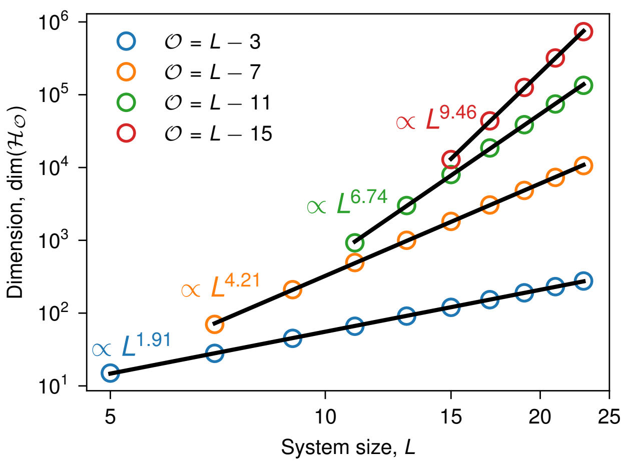

Supplementary Figure 8. **Hilbert space dimension** of sectors with constant $\mathcal{O} = L - m$ for $m \in \{3, 7, 11, 15\}$. These sectors correspond to initial conditions with 1, 2, 3, and 4, $|1\rangle$'s that are well separated by $|0\rangle$'s, respectively. Black lines depict power law fits.

the number of occupied basis states saturates the bound, with the two exceptions being $L = 21$ and $L = 23$. Supplementary Figure 9b shows the total fraction of the scaling bound occupied at *some* point along the first 30 cycles (colors correspond to those in panel a). Supplementary Figure 9b corroborates that the $\dim(\mathcal{H}_{\mathcal{O}})$ scaling bound is always saturated during at least one cycle in the first 30 cycles, with the exceptions again being $L = 21$ and $L = 23$, which reach $\approx 99\%$ and $\approx 98\%$ saturation, respectively.

SUPPLEMENTARY NOTE 5 – MUTUAL INFORMATION MEASURES

Consider a set of z-basis measurements $\{|z\rangle$ s.t. $\in \{0,1\}^{\otimes N}\}$ from an N-qubit (subset of a) quantum processor and associated probabilities, $\{P_z\}$. For any two qubits, q_i and q_j, one can construct a z-basis joint and two marginal probability distributions via

$$p(z_i, z_j) = \sum_{z'} P_{z'}\delta_{z_i,z'_i}\delta_{z_j,z'_j} \tag{19}$$

$$p(z_{i(j)}) = \sum_{z_{j(i)}} p(z_i, z_j). \tag{20}$$

For two classical variables (e.g., bits) z_i and z_j with joint probability distribution $p(z_i, z_j)$ and marginal probability distributions, $p(z_i)$ and $p(z_j)$, the Shannon mutual information is defined in terms of the Shannon entropy of a distribution, $\mathrm{H}(p)$, as (see e.g., Ref. [12])

$$I_{ij} \equiv \mathrm{H}[p(z_i)] + \mathrm{H}[p(z_j)] - \mathrm{H}[p(z_i, z_j)] \tag{21}$$

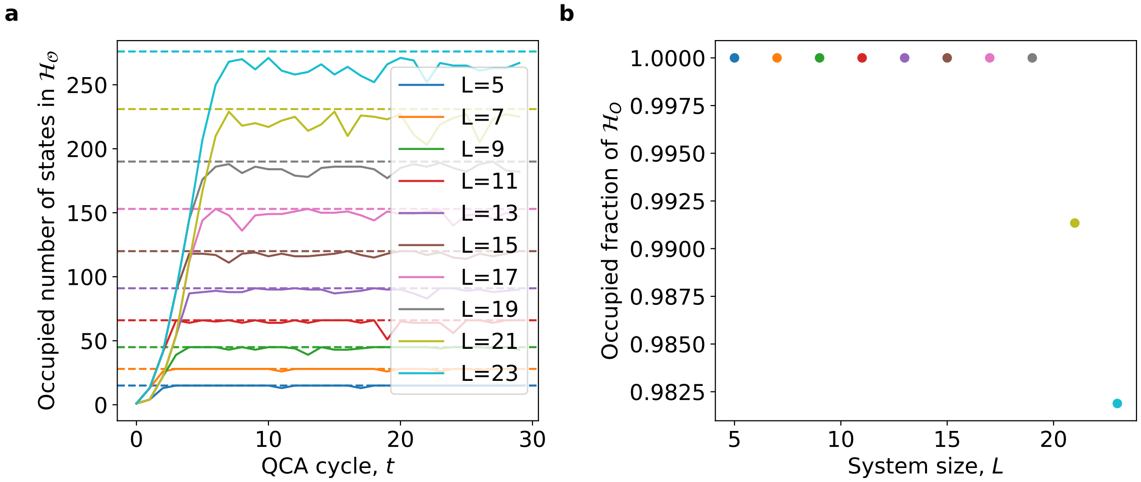

Supplementary Figure 9. **Emulated saturation of protected Hilbert space bound.** **a** Number of basis states involved in superposition as a function of QCA cycle for various system sizes (solid lines). Data generated from classical emulations. Dashed lines represent the $\dim(\mathcal{H}_\mathcal{O}) \sim 0.63\, L^{1.91}$ scaling bound for $\mathcal{O} = L-3$. **b** Corresponding maximally-occupied state as a fraction of scaling bound along the first 30 QCA cycles (colors correspond to those in panel a).

and can be written as

$$I_{ij} \equiv \sum_{z_i=0}^{1}\sum_{z_j=0}^{1} p(z_i,z_j)\log_2 \frac{p(z_i,z_j)}{p(z_i)p(z_j)}. \tag{22}$$

It measures, as a relative entropy, correlations in the variables z_i and z_j. That is, $I_{ij} \geq 0$ and $I_{ij} = 0$ if and only if $p(z_i,z_j) = p(z_i)p(z_j)$.

The quantum mechanical generalization of the classical probability distributions is the reduced density matrix (RDM). For a Hilbert space composed of two subspaces, $\mathcal{H} = \mathcal{H}_A \otimes \mathcal{H}_B$ and a density matrix $\rho \in \mathcal{H}$, the RDM of subspace $\mathcal{H}_A$ is defined as

$$\rho_A \equiv \mathrm{Tr}_B \rho, \tag{23}$$

where the the trace is taken over the degrees of freedom of subsystem $\mathcal{H}_B$. RDMs can also be constructed from few-body quantum observables. For instance, the single-qubit RDM for qubit q_i can be constructed as

$$\rho_i = \frac{1}{2}\sum_{\mu=0}^{3}\langle\sigma_i^\mu\rangle\sigma_i^\mu, \tag{24}$$

where $\sigma_i^0 = 1_i$, $\sigma_i^1 = X_i$, $\sigma_i^2 = Y_i$, and $\sigma_i^3 = Z_i$ are elements of the Pauli algebra, and expectation values are calculated from quantum processor measurements in the appropriate basis [13]. Similarly, a k-qubit RDM can be calculated as

$$\rho_{i_1\ldots i_k} = \frac{1}{2^k}\sum_{\mu_1,\ldots,\mu_k=0}^{3}\langle\sigma_{i_1}^{\mu_1}\otimes\ldots\otimes\sigma_{i_k}^{\mu_k}\rangle\sigma_{i_1}^{\mu_1}\otimes\ldots\otimes\sigma_{i_k}^{\mu_k}. \tag{25}$$

Expectation values of long Pauli strings can be efficiently calculated via schema such as quantum overlapping tomography [14]. With an RDM in hand, the von Neumann entropy is defined as

$$S(\rho) \equiv -\mathrm{Tr}\rho\log_2\rho \tag{26}$$

and the von Neumann mutual information between two qubits follows similarly

$$I_{ij}^{\mathrm{vN}} \equiv S(\rho_i) + S(\rho_j) - S(\rho_{ij}). \tag{27}$$

Calculation of I_{ij}^{vN} therefore requires measurements of observables, such as $\langle X_i Z_j\rangle$, in bases other than the z-basis. However, the dynamical invariant introduced in the main text, $\mathcal{O} = \sum_{i=0}^{L} Z_i Z_{i+1}$, can only be used to post-select for errors when measurement is performed in the z-basis. As such, we choose to use Shannon mutual information for the calculation of complex network measures.

As validation for this choice, we numerically emulate $L = 19$ qubits initialized with a single-bit flip and evolving under T_6 for 10,000 cycles. At each cycle we calculate the relative distance between Shannon- and von Neumann-based network measure, $f \in \{\mathcal{C}, \ell, g_i\}$, as $[f(I) - f(I^{\mathrm{vN}})]/f(I^{\mathrm{vN}})$ and relative Frobenius distance between the two mutual information measures $\|I - I^{\mathrm{vN}}\|_{\mathrm{F}}/\|I^{\mathrm{vN}}\|_{\mathrm{F}}$, where the Frobenius norm of a matrix M with elements M_{ij} is defined as

$$\|M\|_{\mathrm{F}} = \sqrt{\sum_{i,j=0}^{L-1}|M_{ij}|^2}. \tag{28}$$

Supplementary Figure 10a shows a direct comparison of the Shannon- (blue) and von Neumann- (gold) based mutual information network clustering over the first 100

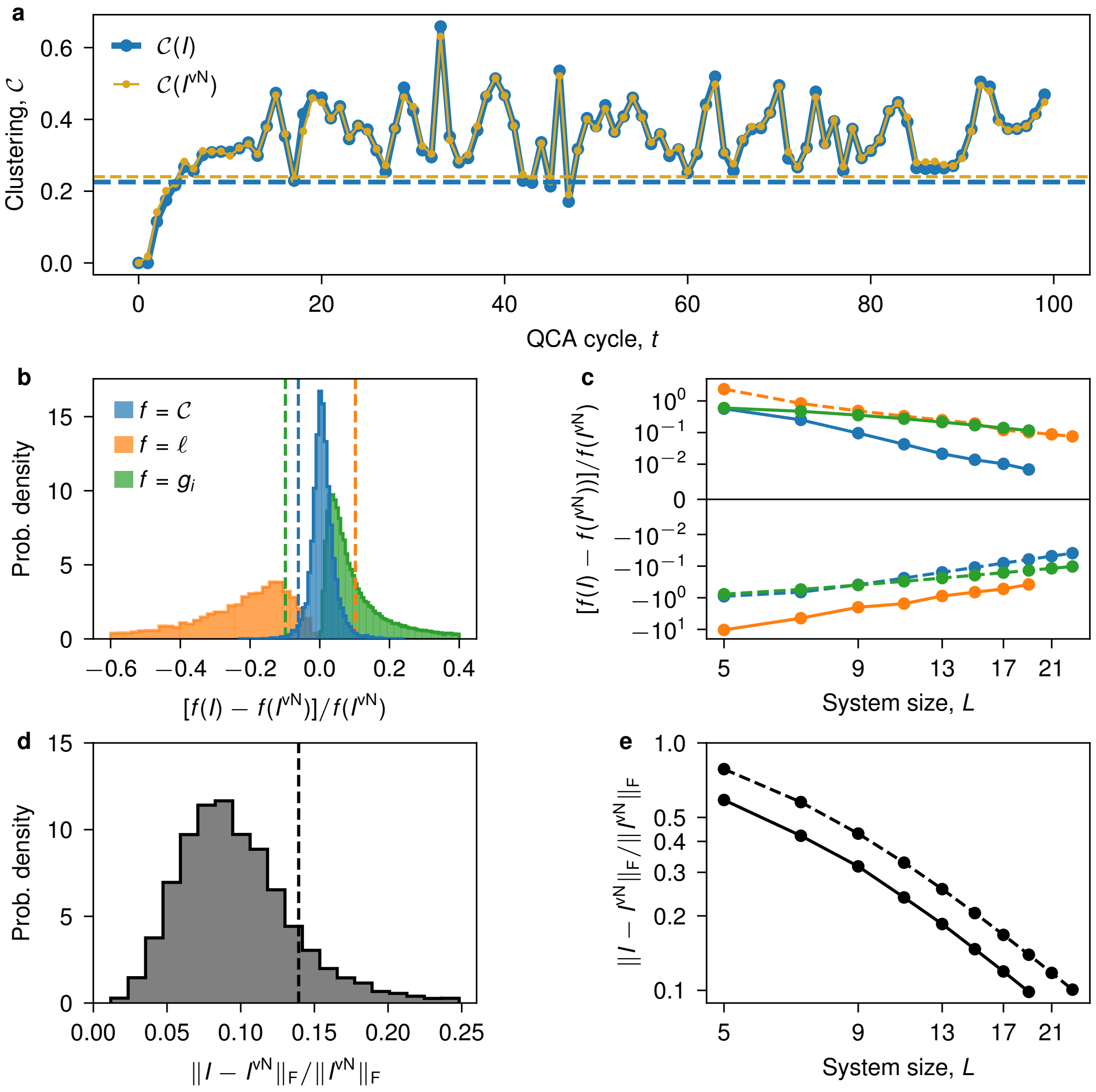

Supplementary Figure 10. **Comparing Shannon and von Neumann mutual information by classical emulation.** **a** Shannon- (blue) and von Neumann- (gold) based mutual information network clustering for 100 QCA cycles and $L = 19$ qubits are in visual agreement. The dashed lines represent the post-selected incoherent uniform random state's clustering for Shannon- and von Neumann-based mutual information. **b** Distribution of relative differences between Shannon- and von Neumann-based network measures across 10,000 QCA cycles and for $L = 19$ qubits. The dashed lines represent the relative differences between network measures for the post-selected incoherent uniform random state. **c** As a function of system size, the mean relative differences between network measures over 10,000 cycles (solid lines) and post selected incoherent uniform random states (dashed lines) tend towards zero. Colors are as in panel b. The 10,000 cycle emulations terminate at $L = 19$ due to computational cost. **d-e** As in panels b-c, but instead of relative differences of network measures, we examine the relative Frobenius distance between Shannon- and von Neumann-based mutual information matrices.

QCA cycles for $L = 19$. Dashed lines represent the corresponding clustering values for post-selected incoherent uniform randomness. Visually, it is clear that the two different mutual information metrics lead to very similar clustering values both for the emulated QCA and post-selected incoherent uniform randomness. In particular, the slight shifts that are observed are insufficient to erase the existence of the coherence windows between $t \sim 4$ and $t \sim 12$ established in Fig. 3 in the main text. Supplementary Figure 10b quantifies and extends this observation with respect to all three complex network measures discussed in the main text: it shows the distribution of relative differences between Shannon- and von Neumann-based mutual information network measures across 10,000 QCA cycles for $L = 19$. Histogram curves represent QCA data and vertical dashed lines represent the corresponding differences for post-selected incoherent uniform randomness. Clustering is the most similar between Shannon- and von Neumann-based mutual information (blue histogram, mean relative difference 0.8%,

median 0.7%). Path length (gold histogram) exhibits a mean relative difference of −38%, (median −21%) while that of node strength (green histogram) is 12% (median 7%). For the $L = 19$ post-selected incoherent uniform random state, the relative difference Shannon- and von Neumann-based clustering is −6.1% while that of path length is 10% and average node strength is −14%. Supplementary Figure 10c shows that as a function of system size, the mean relative differences between network measures over 10,000 cycles (solid lines) and post selected incoherent uniform random states (dashed lines) tend towards zero roughly as a power law. Colors are as in panel b. The 10,000 cycle emulations terminate at $L = 19$ due to computational cost. Hence, we conclude not only that Shannon mutual information serves as an effective proxy for von Neumann mutual information in the calculation of complex network measures in our regime of interest, $L \in \{5, 7, \ldots, 23\}$, but that it would become an even more effective proxy in the large L limit. As in Supplementary Figure 10b-c, but instead of relative differences of network measures, Supplementary Figure 10d-e shows the relative Frobenius distance between Shannon- and von Neumann-based mutual information matrices directly. The mean relative Frobenius distance over 10,000 QCA cycles is 10% (median 9%) while that of post-selected randomness is 14%. From the above analysis, we conclude that Shannon mutual information is a reliable proxy for von Neumann mutual information at the level of a few to several percent for $L = 19$ and that the relative difference between the two types of mutual information tends monotonically towards zero as L increases.

Finally, aside from the requirement to perform post-selection for error mitigation purposes using the operator $\mathcal{O}$ in Eq. 10, we justify the use of the computational z-basis as the relevant measurement basis in which to construct the Shannon mutual information as opposed to the y-basis or x-basis (which, for instance, is related to the z-basis by L-qubit Hadamard transform). First, we note that measurement in the y-basis would yield no Shannon mutual information between any pairs of qubits for the initial conditions considered in this work. This is because all qubits are either initialized in the $|0\rangle$ or $|1\rangle$ state and a series of Hadamard activation unitaries will never move the dynamics of states so initialized out of the xz-plane of a qubit's Bloch sphere. It remains to show that the choice of z-basis is at least as sensible as choosing the x-basis. Supplementary Figure 11 shows the clustering coefficient for Shannon mutual information networks calculated based on measurements in the x-basis (blue curves) and z-basis (orange curves) as a function of QCA cycle for three different system sizes, $L \in \{15, 17, 19\}$. While the z-basis Shannon mutual information network clustering tracks the von Neumann mutual information network clustering (Supplementary Figure 10a) closely, the x-basis Shannon mutual information network clustering fluctuates only slightly from the Shannon mutual information network clustering of incoherent uniform randomness (black dotted line), which is a basis-independent quantity, for the system sizes shown. This is intuitive, since the amount of correlation structure (in this case clustering) between degrees of freedom in one measurement basis comes at the expense of correlation structure carried in a different basis if the total degree of correlations is bounded by the von Neumann mutual information. Taken together with the rest of the evidence presented in Supplementary Figure 10, it is clear that z-basis Shannon mutual information is the correct network quantity with which to establish the existence of correlations with small-world structure.

SUPPLEMENTARY NOTE 6 – COMPLEX NETWORK MEASURES

Clustering Coefficient

In order to establish the small-world character of the T_6 QCA's mutual information network, we focus on three canonical complex network measures: clustering, path length, and node strength distribution. Intuitively, one can view the mutual information matrix I_{ij}, as an adjacency matrix in analogy with that of a transportation network. For example, the economic activities of two cities are likely to be more tightly intertwined if high-speed rail (large adjacency weight) connects them rather than a rutted dirt track (low adjacency weight). Similarly, the dynamics of two qubits will be more strongly correlated if they share more mutual information.

Continuing with this analogy, the clustering coefficient gauges how locally traversable a network is. Suppose Alice is attempting to reach City C from City A, but there only exist rail lines from City A to City B and from City B to City C, but no lines exist, or perhaps just a dirt track exists, between City A and City C. Locally, this is an inefficient network to traverse since Alice either has to travel through City B else has to take the dirt track to get between Cities A and C. A highly traversable network at the local scale also implies high network transitivity; that is, if Cities A and B are well-connected and Cities B and C are well-connected, then so should be cities A and C. The *global clustering coefficient* assesses this local transitivity in a manner that averages over the entire network. At a particular cycle depth, t, it is defined as

$$\mathcal{C} \equiv \frac{\mathrm{Tr}[I^3]}{\sum_{i \neq j=1}^{L} [I^2]_{ij}}. \tag{29}$$

The numerator of Eq. 29 counts the weighted number of closed triangles (node triplets) in the network, while the denominator is the weighted number of length-2 paths in the network, that is, the weighted number of potentially closed triangles. In the main text, we show that

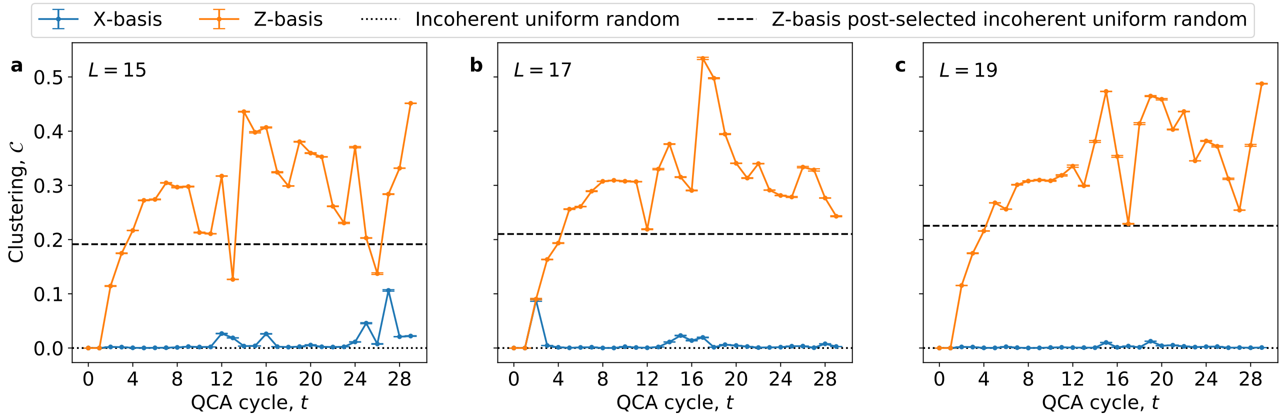

Supplementary Figure 11. **Comparing clustering calculated via x-basis and $z-$basis Shannon mutual information by classical emulation.** Clustering coefficient calculated for the Shannon mutual information network based on x-basis (blue) and z-basis (orange) measurements as a function of the first 30 QCA cycles for three different system sizes, $L = 15$ (**a**), $L = 17$ (**b**), and $L = 19$ (**c**). All data is from classical emulation. The dotted black lines represent the clustering of incoherent uniform randomness, which is measurement basis independent. Dashed black lines show, for reference, the $z-$basis Shannon mutual information-based clustering of post-selected incoherent uniform randomness.

the Goldilocks rule T_6 exhibits sizeable clustering, much larger than the post-selected uniform random state. The clustering grows with system size within the Weber processor coherence window, indicating experimental formation of a series of locally traversable networks.

Average Shortest Path Length

A network's *average shortest path length* measures the extent to which it is globally traversable. In the transportation network analogy, a network of cities (e.g., Atlanta, Nashville, Indianapolis, Detroit, Pittsburgh, Baltimore, Raleigh, and Charlotte) laid out in a one-dimensional ring topology but only connected to their nearest neighbors (Atlanta and Indianapolis for Nashville) and next-nearest neighbors (Charlotte and Detroit for Nashville) via high-speed rail lines would have very high local traversability, i.e., transitivity. However, it would take a relatively long time to traverse between antipodes of the ring (e.g., Nashville to Baltimore) and thus those cities' economic activities might be less correlated. From the standpoint of network weights, a large mutual information between two nodes should contribute to shortening path length, such as a rail line that could carry more passengers might, while small mutual information should increase the path length by virtue of its low traversability, like the dirt track. We therefore define the network-averaged weighted path length for a particular cycle depth as

$$\begin{aligned} \ell &\equiv \frac{1}{L(L-1)} \sum_{i\neq j=1}^{L} d_{ij}, \\ d_{ij} &= \min_{p_{ij}\in\mathcal{P}_{ij}} \sum_{\langle k,l\rangle\in p_{ij}} 1/I_{kl}, \end{aligned} \tag{30}$$

where d_{ij} is the minimum distance between nodes (i.e., qubits) q_i and q_j, the sum in the first line runs over all pairs of qubits, and $L(L-1)$ is the number of pairs in the network. The sum in the second line runs over all edges ($\langle k,l\rangle$) in a particular path (p_{ij}) between nodes q_i and q_j and the minimum is taken over all possible paths ($\mathcal{P}_{ij} = \{p_{ij}\}$) between the two nodes. As discussed, we use $1/I_{kl}$ as weights in the summand under the intuition that edges with large mutual information are traversed easily while small mutual information values should be detrimental to short path length, consistent with [15].

Node-Strength Distribution

The last complex network measure we consider is the *node-strength distribution*. In the transportation network analogy, an efficiently traversable network will have many nodes like Grand Central Station (termed *hubs*) that have many, strong connections to other nodes as well as some nodes that have few or weak connections, like Golden, Colorado, whose servicing light rail line terminates three miles short of the center of town. Mathematically, this translates into a broad, flat node-strength distribution. We calculate a size-invariant (un-normalized)

node-strength distribution,

$$P[g_i/(L-1)] = \text{hist}\left[\frac{1}{L-1}\sum_j I_{ij}\right] \quad (31)$$

Practically, Eq. 31 is evaluated by aggregating all size-scaled values of $g_i/(L-1) = \sum_j I_{ij}/(L-1)$ across all system sizes, $L \in \{5, 7, \ldots, 23\}$ into a set which is then histogrammed. The resulting distribution is increasingly biased towards low node strength as the network approaches randomness. Small-world networks have broad, flat distributions, while distributions that are more sharply-peaked around smaller ranges of values indicate increasing non-random regularity in the network.

Relationship to Prior Complex Mutual Information Network Analyses

In wireless communications networks [16], where nodes are connected by channels with limited bandwidth, or brain networks, where functional connectivity is distinct from spatial connectivity [17], network measures carry similar meaning as the mutual information networks herein. For instance, a communications network with higher channel capacity (upper bound on amount of information transmissible between two nodes) should have a shorter path length between the nodes, because it is easier for information to move between the nodes. Similarly, in a functional brain network described by mutual information, nodes (i.e., functional regions) within a pair that share more mutual information will affect one another's state to a greater extent. Hence, path length should also be lower in this instance. The intuition behind these examples is that thresholding a network with a minimum channel capacity or mutual information will only leave edges between strongly-linked nodes. Weak links will be removed. Hence, if only weak links exist between nodes prior to thresholding, they will be removed, contributing to a longer path length– which after thresholding is calculated just by counting the number of edges traversed– between the nodes. Similar, common meanings also exist for clustering and node strength distribution between QCA, communications, and functional brain networks.

SUPPLEMENTARY NOTE 7 – ESTABLISHING THE COHERENCE WINDOW

As discussed in the main text, our Goldilocks QCA circuits, when aided by post-selection, generate mutual information complex network observables that exhibit structure in excess of post-selected incoherent uniform randomness. Supplementary Figure 12 shows the clustering coefficient calculated from the post-selected data generated from the Weber processor (green data points),

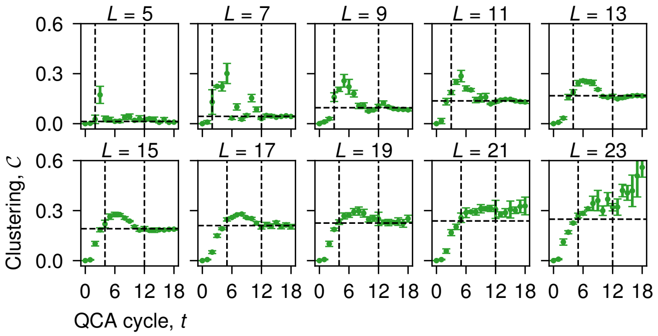

Supplementary Figure 12. **Establishing the coherence window.** Clustering coefficient calculated from the post-selected data generated from the Weber processor (green data points), as a function of QCA cycle and for all system sizes simulated (one in each panel), $L \in \{5, 7, \ldots, 23\}$. Error bars: one standard deviation from the mean on four different qubit chains. Horizontal dashed lines: clustering coefficient for post-selected incoherent uniform randomness, based on the QCA initial condition of a single $|1\rangle$ initialized in a chain of L qubits. Vertical dashed lines: beginning and end of the coherence window in each subplot.

as a function of QCA cycle and for all system sizes simulated, $L \in \{5, 7, \ldots, 23\}$. Over the first few cycles, the clustering coefficient climbs from zero until it crosses the horizontal dashed line in each panel, which is the value of the clustering coefficient for the incoherent uniform random state subject to the same post-selection procedure as the QCA data from Weber. The left-most vertical dashed line demarcates this point, which is the QCA cycle at which the post-selected experimental clustering becomes larger than post-selected randomness. In all cases, the post-selected experimental clustering continues to climb for a few cycles until decoherence mechanisms begin to degrade the data in excess of what post-selection can mitigate for, typically at $t \approx 8$. After a few more cycles, the post-selected experimental clustering drops to the clustering value of post-selected incoherent uniform randomness and remains there for the rest of the simulation. The right-most vertical dashed line demarcates this point, which occurs at $t \approx 12$ roughly independent of system size. For each system size, the region between the two vertical dashed lines is where the post-selected experimental clustering is in excess of post-selected incoherent uniform randomness and is therefore the *coherence window* over which we calculate the cycle-averaged complex network observables. We remark that after the end of the coherence window, $t \approx 12$, for $L = 21$ and $L = 23$ the post-selected experimental clustering fails to return to the value associated with post-selected incoherent uniform randomness. We do not regard this as an extension of the coherence window. Rather, inspection of Supplementary Figure 7 shows that after $t \approx 12$, the 21-qubit chains are retaining fewer than roughly 100 of the 100,000 initial measurements while the 23-qubit chains

are retaining fewer than about 10 of the initial 100,000 measurements. As such, error bars become significant and calculation of observables after $t \approx 12$ in each case becomes unreliable.

SUPPLEMENTARY NOTE 8 – EFFECT OF HIGHER PRODUCT STATE FILLING

Throughout the main text, we have focused on a particular initial condition for our Goldilocks QCA circuits, namely $|0\dots010\dots0\rangle$, a single central bit flip in a chain of L qubits. This was done so as to be able to perform finite size scaling analyses of complex network measures in a sensible and well-controlled manner, as well as the close analogy to a standard initial condition for elementary classical cellular automata. Moreover, it was previously shown that the ability of Goldilocks QCA to generate physical complexity is largely insensitive to initial conditions [3]. However, it is an important question as to whether our experimental protocol, including post-selection, for effectively generating coherent small-world mutual information networks generalizes beyond the particular instance of the single bit-flip initialization.

Supplementary Figure 13 shows the effect of increasing the number of isolated bit flips in the initial state on clustering dynamics of a 17-qubit chain simulated on Google's 23-qubit Rainbow processor. In particular, the Supplementary Figure 13a corresponds to the initial condition $|00000100000100000\rangle$, Supplementary Figure 13b corresponds to the initial condition $|00010000100001000\rangle$ and Supplementary Figure 13c corresponds to the initial condition $|00100010001000100\rangle$, i.e., equally spaced $|1\rangle$'s. We observe the following. First, the long-time average clustering values of the emulated data fall as the number of initial bit flips increase. Second, the raw clustering follows the expected behavior in all the panels, rising briefly before decaying down to incoherent uniform randomness at around $t \approx 12$. Third and most importantly however, we note that increasing the number of initial bit flips degrades the ability of post-selection to correct for errors. While two initial bit flips (Supplementary Figure 13a) results in post-selected experimental clustering that follows the emulated curve relatively closely until $t \approx 4$ when it begins to degrade, three bit flips (Supplementary Figure 13b) results in agreement out to only $t \approx 2$, and four initial bit flips (Supplementary Figure 13c) sees immediate disagreement between emulation and post-selected experiment after $t \approx 1$. In fact, for four initial bit flips, there is no QCA cycle for which post-selected experimental clustering is meaningfully improved over raw experimental clustering during the coherence window. This indicates that while our experimental protocol, including post-selection, is most appropriately applied at very low initial bit flip filling, it struggles to produce emergent complexity in the face of noise at higher filling fractions. This is likely because at higher initial isolated bit-flip filling, there are more error processes that cause amplitude renormalization but still keep the state vector in the protected sector of Hilbert space. That is, there are more locations in a higher-filling bit string where an erroneous bit flip will nonetheless result in domain wall conservation.

SUPPLEMENTARY NOTE 9 – COMMENT ON SIMULATION PLATFORM CHOICE

Here, we comment briefly on our choice of simulation platform for the experimental realization of QCA. First, we justify the use of a universal, gate-model processor against an analog quantum simulator. Chains of Rydberg atoms up to 51 and trapped ions up to 53 sites long have been used simulate the many-body dynamics of kinetically-constrained (sometimes called PXP [18]) and quantum Ising-type spin models out to microseconds and milliseconds long, respectively [19, 20]. These are the existing experiments that most closely approximate ours on the analog simulation side of the field. While these experiments exhibit larger qubit counts and, in some instances, better coherence properties, analog quantum simulators are highly-constrained in the systems they can simulate by the native degrees of freedom and interactions of the underlying simulator. That is, while particular analog simulation platforms may be appropriate for simulating particular quantum cellular automata, it would be very difficult to design one as a general-purpose QCA simulator. This reflects the need for universal, gate-model machines writ large. As such, though it is required in our experiment to combat higher noise levels than those found in analog simulators through a variety of techniques, some of which thereby limiting the observables we are able to calculate, our approach to QCA simulation is generalizable not just to other QCA in one-dimension, but to those of higher dimension and greater connectivity as well, given sufficiently improved noise characteristics of the underlying quantum processor.

Second, having addressed the issue of analog versus digital quantum simulation, we remark upon our particular choice of digital platform. There currently exist various approaches to constructing gate-model quantum computers, among them superconducting, trapped ion, neutral atom, photonic, and topological qubit-based processors. Each of these approaches involves design trade-offs. At the time of performing our experiment, the large majority of cloud-accessible gate-model quantum computers were either trapped ion (e.g., Quantinuum, IonQ) or superconducting machines (e.g., Google, Rigetti, IBM). The capability of a given quantum processor involves trade-offs in terms of qubit count, qubit connectivity, decoherence times (relative to gate execution times), native gate expressiveness, and gate and state-

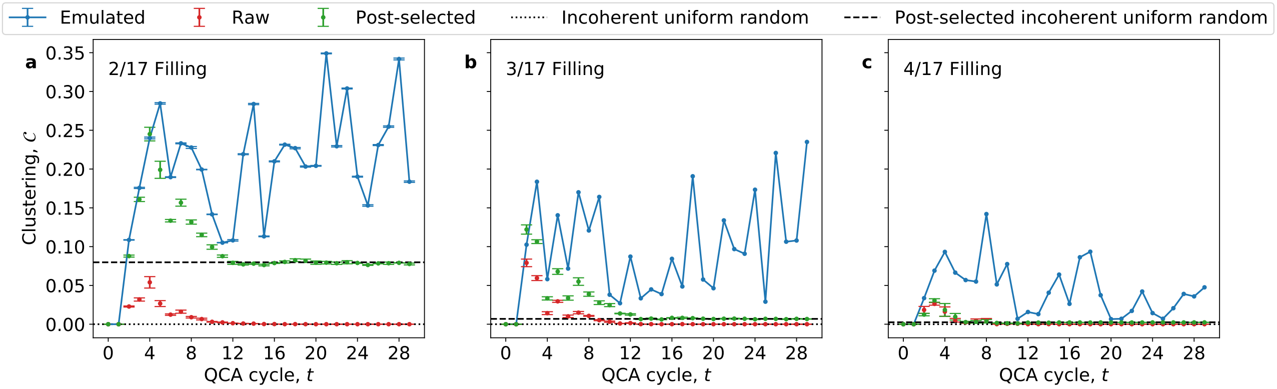

Supplementary Figure 13. **Effect of higher product state filling.** Clustering coefficient as a function of QCA cycle for three different numbers of isolated $|1\rangle$s in the initial product state, 2 (**a**), 3 (**b**), and 4 (**c**), for $L = 17$ on the Rainbow processor (blue curves: numerical emulation; red points: Rainbow data without post-selection; green points: with post-selection). Error bars are one standard deviation in $\mathcal{C}$ over four different qubit chains. Black dashed lines represent the clustering of the incoherent uniform random state, subject to post-selection on the appropriate eigenvalues of $\mathcal{O}$ corresponding to the three different initial conditions: 10 $(L-7)$, 6 $(L-11)$, and 2 $(L-15)$.

preparation and measurement error rates. While trapped ion two-qubit error rates can be somewhat lower than superconducting error rates– e.g., $\sim 0.3\%$ for Quantinuum's H1.1 processor– and are fully-connected due to the ability to shuttle ions, cloud accessible trapped ion processors remain rather size-limited with respect to superconducting processors [21]. For example, at the time our experiment was performed, Quantinuum's cloud-accessible processor could load up to 12 ions and IonQ's processor could load up to 11 ions. By comparison, we have simulated QCA as large as 23 qubits long. Moreover, we did not require all-to-all connectivity for our QCA circuits since all interactions were nearest-neighbor. We also note that our employment of the Floquet calibration technique allowed for the additional suppression of two-qubit gate error in our system beyond the $\sim 1.4\%$ rate determined by cross-entropy benchmarking. As for superconducting processors, the Rainbow and Weber processors on which we ran our experiments are at least as performant as those offered by other superconducting processor vendors such as IBM or Rigetti. In summary, it is unlikely that running our QCA circuits on a different current-generation, gate-model quantum processor would have resulted in a positive step-change in terms of fidelity, although we would be fascinated to see our results repeated or improved upon on a different architecture.

---

* eric.jones@coldquanta.com
† ekapit@mines.edu
‡ lcarr@mines.edu

[1] Arrighi, P. An overview of quantum cellular automata. *Natural Computing* **18**, 885–899 (2019).
[2] Farrelly, T. A review of quantum cellular automata. *Quantum* **4**, 368 (2020).
[3] Hillberry, L. E. *et al.* Entangled quantum cellular automata, physical complexity, and goldilocks rules. *Quantum Science and Technology* **6**, 045017 (2021). URL `http://iopscience.iop.org/article/10.1088/2058-9565/ac1c41`.
[4] Ritort, F. & Sollich, P. Glassy dynamics of kinetically constrained models. *Advances in Physics* **52**, 219–342 (2003).
[5] Subrahmanyam, V. Domain wall dynamics of the ising chain in a transverse field. *Physical Review B* **68**, 212407 (2003). URL `https://link.aps.org/doi/10.1103/PhysRevB.68.212407`.
[6] Arute, F. *et al.* Quantum supremacy using a programmable superconducting processor. *Nature* **574**, 505–510 (2019).
[7] AI, G. Q. Quantum computer data sheet. `https://quantumai.google/hardware/datasheet/weber.pdf` (2021).
[8] Developers, C. Cirq (2021). URL `https://doi.org/10.5281/zenodo.5182845`. See full list of authors on Github: https://github.com/quantumlib/Cirq/graphs/contributors.
[9] Arute, F. *et al.* Observation of separated dynamics of charge and spin in the fermi-hubbard model. *Preprint at https://arxiv.org/abs/2010.07965* (2020).
[10] Neill, C. *et al.* Accurately computing the electronic properties of a quantum ring. *Nature* **594**, 508–512 (2021).
[11] Aaronson, S. & Chen, L. Complexity-theoretic foundations of quantum supremacy experiments. In *Proceedings of the 32nd Computational Complexity Conference*, CCC '17 (Schloss Dagstuhl–Leibniz-Zentrum fuer Informatik, Dagstuhl, DEU, 2017).
[12] Nielsen, M. A. & Chuang, I. *Quantum Computation and Quantum Information* (Cambridge University Press, Cambridge, 2000).
[13] Gamel, O. Entangled bloch spheres: Bloch matrix and two-qubit state space. *Physical Review A* **93**, 062320 (2016).

[14] Cotler, J. & Wilczek, F. Quantum overlapping tomography. *Physical Review Letters* **124**, 100401 (2020).

[15] Muldoon, S. F., Bridgeford, E. W. & Bassett, D. S. Small-world propensity and weighted brain networks. *Scientific Reports* **6**, 1–13 (2016).

[16] Urgaonkar, R. & Neely, M. J. Optimal routing with mutual information accumulation in wireless networks. *IEEE Journal on Selected Areas in Communications* **30**, 1730–1737 (2012).

[17] Bullmore, E. & Sporns, O. Complex brain networks: graph theoretical analysis of structural and functional systems. *Nature Reviews Neuroscience* **10**, 186–198 (2009).

[18] Turner, C. J., Michailidis, A. A., Abanin, D. A., Serbyn, M. & Papić, Z. Quantum scarred eigenstates in a rydberg atom chain: Entanglement, breakdown of thermalization, and stability to perturbations. *Physical Review B* **98**, 155134 (2018). URL `https://link.aps.org/doi/10.1103/PhysRevB.98.155134`.

[19] Bernien, H. *et al.* Probing many-body dynamics on a 51-atom quantum simulator. *Nature* **551**, 579–584 (2017).

[20] Zhang, J. *et al.* Observation of a many-body dynamical phase transition with a 53-qubit quantum simulator. *Nature* **551**, 601–604 (2017).

[21] Lubinski, T. *et al.* Application-oriented performance benchmarks for quantum computing. *Preprint at https://arxiv.org/abs/2110.03137* (2021).
